# Application of a self-enhancing classification method to electromyography pattern recognition for multifunctional prosthesis control

**DOI:** 10.1186/1743-0003-10-44

**Published:** 2013-05-01

**Authors:** Xinpu Chen, Dingguo Zhang, Xiangyang Zhu

**Affiliations:** 1 State Key Laboratory of Mechanical System and Vibration, School of Mechanical Engineering, Shanghai Jiao Tong University, Shanghai 200240, China

**Keywords:** Electromyography (EMG), Myoelectric control, Pattern recognition, Self-enhancing classification, Prostheses

## Abstract

**Background:**

The nonstationary property of electromyography (EMG) signals usually makes the pattern recognition (PR) based methods ineffective after some time in practical application for multinational prosthesis. The conventional EMG PR, which is accomplished in two separate steps: training and testing, ignores the mismatch between training and testing conditions and often discards the useful information in testing dataset.

**Method:**

This paper presents a novel self-enhancing approach to improve the classification performance of the electromyography (EMG) pattern recognition (PR). The proposed self-enhancing method incorporates the knowledge beyond the training condition to the classifiers from the testing data. The widely-used linear discriminant analysis (LDA) and quadratic discriminant analysis (QDA) are extended to self-enhancing LDA (SELDA) and self-enhancing QDA (SEQDA) by continuously updating their model parameters such as the class mean vectors, the class covariances and the pooled covariance. Autoregressive (AR) and Fourier-derived cepstral (FC) features are adopted. Experimental data in two different protocols are used to evaluate performance of the proposed methods in short-term and long-term application respectively.

**Results:**

In protocol of short-term EMG, based on AR and FC, the recognition accuracy of SEQDA and SELDA is 2.2% and 1.6% higher than conventional that of QDA and LDA respectively. The mean results of SEQDA(C) and SEQDA (M) are improved by 2.2% and 0.75% for AR, and 1.99% and 1.1% for FC respectively when compared to QDA. The mean results of SELDA(C) and SELDA (M) are improved by 0.48% and 1.55% for AR, and 0.67% and 1.22% for FC when compared to LDA. In protocol of long-term EMG, the mean result of SEQDA is 3.15% better than that of QDA.

**Conclusion:**

The experimental results show that the self-enhancing classifiers significantly outperform the original versions using both AR and FC coefficient feature sets. The performance of SEQDA is superior to SELDA. In addition, preliminary study on long-term EMG data is conducted to verify the performance of SEQDA.

## Introduction

Surface electromyogram (EMG) signal is a noninvasive measurement and contains rich information associated with the muscle electrical activities. It is considered to be an important input for the control of electrically powered prostheses, referred to as myoelectric control [1]. Conventional myoelectric control systems enable the amputees to operate a single device such as a hand or a wrist [2], simply based on amplitude decoding of the EMG signal recorded from the separable forearm muscles. The early myoelectric controllers can only operate in an on-off mode to control electrically powered hands with open-close functions [3]. Controlling a multi-degree prosthetic hand requires more sophisticated technique for decoding of different muscle states from the recorded EMG [4].

To increase the number of motion classes, much attention has been drawn to a pattern-recognition (PR)-based approach to the myoelectric control of multifunctional prostheses in last two decades. Unlike the conventional EMG decoding method that assigns each function to a specific control muscle, the PR-based approach extracts useful information from several EMG channels to form a feature vector and maps it to a motion class, maximizing the separability between each motion. Several types of EMG PR systems are introduced to fulfill the multifunctional prothesis control [2,5-10].

The feature extraction and classifier design are the major components of PR-based control strategy. The performance of EMG PR is mainly evaluated by the classification accuracy. Various EMG feature sets have been employed to extract the most discriminant information for improving the classification accuracy. The feature extraction methods include autoregressive (AR) model [11], multivariate AR model [12], time domain statistics [2,13], root mean square (RMS) [14], higher-order statistics [15], cepstral coefficients [16], time-frequency representation [17,18] and EMG preprocessing method, e.g. the individual principal component analysis (iPCA) [8]. To achieve a high classification accuracy, researchers have extensively explored different types of classifiers, such as MLP [2], LDA [7,19], Gaussian Mixture Model [9], hidden Markov model [6], support vector machine [10], fuzzy logic [20], K-nearest neighbor classifier [21] and unsupervised clustering [22]. In addition, due to the large number of EMG channels [23] and high dimensionality of feature set, feature selection and feature projection methods such as sequential feedforward selection [8,23], PCA [17] and uncorrelated linear discriminant analysis [24] are used to transform the EMG features to a lower dimensional subspace.

Usually, a successful classifier of EMG PR method is accomplished by two separate parts: (1) training step that aims to train the classification model from the knowledge of training data and (2) testing step that simulates the situation in real-world application and evaluates the classification performance using the testing data. However, the training EMG data are normally acquired at one time during a short period, and the contained information is limited, so they cannot be representative to the data of whole temporal span in application period including testing step. In real-world application, if an EMG classifier is trained well for a specific amputee, the amputee can control the prothetic hand well at the early stage, but the performance is degraded as the time moves on. This phenomenon is very common, and it is mainly because of the nonstationary property of EMG signals. The possible EMG variation is contributed to these factors such as electrodes condition, muscle fatigue, sweating and so on [25-27]. It is a big problem hindering the commercialization of advanced myoelectric controlled prosthetic hand that was developed in laboratory environment. Therefore, we plan to make further exploration in the testing stage since it simulates the real application situation, and expect to develop a kind of robust or adaptive classifier. In previous research, the training and testing steps are two independent processes. When there exists mismatch between training and testing conditions, the performance of the EMG PR might deteriorate, i.e. the classification accuracy decreases. Enlarging the EMG recordings in training step that contain more information may be a possible solution, but it is a time-consuming task and can give additional burden to the users. So we are inspired to retrain the classifier with the testing data in addition to the training data, which perhaps can alleviate the mismatch problem. In previous research, the parameters of original classifiers, e.g. the mean vector and pooled covariance in LDA, are estimated from the training set only. We believe using more available data to train classifiers can lead to more accurate and stable parameter estimation that is close to the true sampling distribution. Exploiting information in testing dataset is a possible way to enlarge the data pool for training and further increases the recognition accuracy of classifiers.

In order to guarantee the stable performance of the continuous EMG PR in view of above remarks, the idea of self-enhancing classifiers is presented in this paper. As far as we have known, few previous works in myoelectric pattern recognition focus on the classifier adaptation, especially in designing an adaptation procedure for the continuous classification.

In this paper, we extend the LDA and quadratic discriminant (QDA) classifiers to self-enhancing versions since LDA is a popular classifier used widely in many previous studies. It is easy to use and its classification performance is not inferior to other complicated classifiers [28]. The remainders of the paper are organized as follows. Section ‘Method’ explains the methods applied in the EMG signal classification process, including data acquisition, feature extraction, and proposed self-enhancing LDA and QDA (SELDA and SEQDA) classifiers. Section ‘Experiment results’ provides the experimental results. Section ‘Discussion’ is the discussion. Finally, conclusions are presented in Section ‘Conclusion’.

## Method

**Figure 1:**
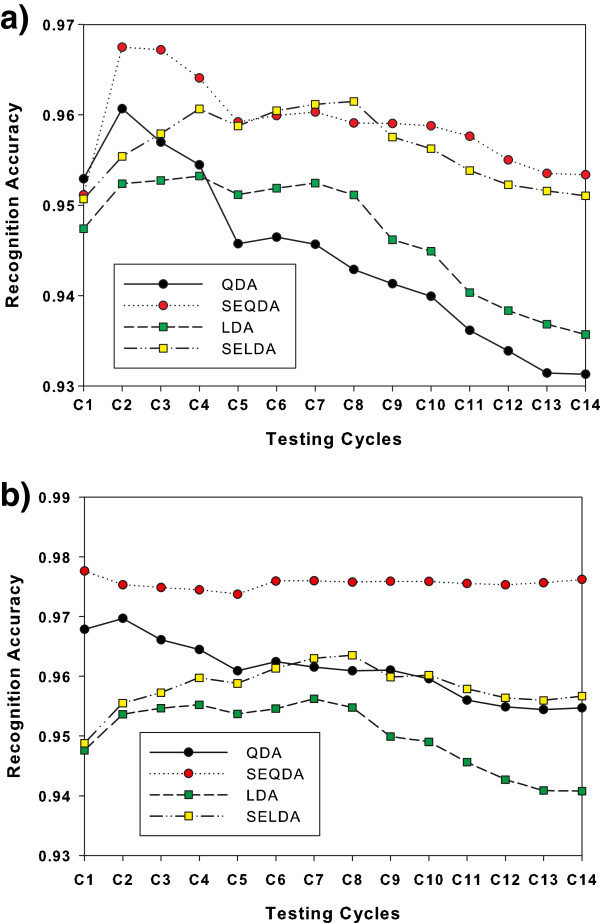
**Block diagram of the proposed self-enhancing EMG classification scheme.** The red line presents the proposed self-enhancing procedure that works as a feedback to update the classifier.

The traditional process of EMG PR method generally contains segmentation, feature extraction, and classification. The decision streams are finally generated for the motion controller. A self-enhancing mechanism is added to the traditional process in this work, and Figure 1 illustrates the flowchart. The key components will be expounded in the following parts.

### Segmentation

The *N*-sample analysis window, which is used to estimate the feature, segments the raw EMG signal and slides with *m*-sample window increment. The procedures of the feature extraction and classification are completed in the window-increment intervals. The continuous classifier sequentially produces a stream of prediction decision for each analysis window. The self-enhance classifier is initiated by training set and then updated its model using the classified continuous EMG data. The self-enhancing step works as a feedback process to the classifier when assessing the classifier in testing step or applying it in real-world application. To easily fit the manner of continuous EMG PR, the self-enhancing algorithm adopts an incremental mode (updating window by window). The parameters of classifiers are continuously adjusted to each new-coming testing data. The data is then thrown away after completing the classifier updating. Namely, the incremental self-enhancing method has the advantage of the small storage requirements. In order to completely evaluate the proposed methods, two protocols are designed for EMG data recording. One is the conventional case, testing data is collected adjacently after the training data measurement. The other case is to let the testing data be collected about 7 hours later after the training data measurement. Generally the EMG data used are collected during a short period (2 ∼3 hours) in previous research, i.e. the data are short-term. Towards the practical application in future, long-term EMG data are more meaningful.

### EMG feature extraction

The surface EMG signal detected during the voluntary contraction resembles stochastic noise due to the variability of MUs (Motor Units) firing rate and recruiting rate. Although EMG signal recording from different motions is a non-stationary process, it has demonstrated that the signal can be assumed to be wide-sense stationary under the 0.5 s analysis windows if the contractions are isotonic and isometric[29]. For the continuous EMG PR, it has no advantages to use the time-scale methods, such as wavelet and wavelet pack [7], to extract EMG features from steady-state signals. Time and frequency analyses are selected to extract the useful features of EMG signal in terms of classification accuracy. Previous studies have shown that the feature set AR + RMS, which respectively describes the amplitude and spectral information of EMG, presents better classification performance than other features [10,28].

The cepstral coefficient is an efficient feature in speech recognition. The AR-derived cepstral coefficient has been applied in EMG PR task and presents good classification performance [16]. Another way of cepstrum coefficients derivation is based on the Fourier spectrum [30]. The discrete cosine transform (DCT) [31] is used for converting fourier spectrum to the meaningful cepstral feature since it can decorrelate the feature and compress spectral information. The Fourier-derived cepstral (FC) is well studied in [32], and it shows better performance compared with other EMG features. The FC coefficients are achieved by two steps: 

1) calculate the energy spectrum using the discrete Fourier transform (FT)

(1) $$X[k]=\overset{N-1}{\underset{n=0}{\sum }}x[n]\;\exp^{{-}_{j}\frac{2\pi }{N}\text{nk}},\;k=0,1,\ldots ,N-1$$

2) calculate FC coefficients from the nonlinear magnitude of the Fourier-spectrum transform directly using DCT

(2) $$\begin{array}{c} FC_{i}=\overset{N-1}{\underset{k=0}{\sum }}Y_{k}\cos(\frac{(k+1/2)(i-1)\pi }{N}),\;i=1,2,\ldots ,N \end{array}$$

where *x*[*n*] is the sEMG signal, *Y*_*k*_=*f*(|*X*[*k*]|) denotes a nonlinear transformation (e.g., logarithm of magnitude) of |*X*[*k*]|, |*X*[*k*]| is the magnitude of Fourier coefficients, and *N* is the number of FC coefficients. In addition, it should be noted that the computation of the FC feature extraction is mainly dependent on the fast FT (FFT) and DCT algorithms and it is computationally efficient.

Since AR and FC have shown superior performance in previous study, they are selected as the EMG feature sets to evaluate the performance of the self-enhancing classifiers proposed in this paper. More details about AR and FC can be found in [11,32], respectively.

### Classifier design

Our improvement is based on two conventional linear and nonlinear classification methods: LDA and QDA. The LDA and QDA classifiers are the Gaussian Maximum-likelihood classification methods based on the Bayes’ rule. LDA has been demonstrated to be suitable for the EMG PR. In addition, LDA and QDA classifiers have no manually specified hyperparameters that significantly affect the generalization performance, thus eliminating trial-and-error approaches such as cross-validation, and the whole classifiers are determined by the training set.

Given an input feature vector *x* for classifiers, the Bayes decision rule shows that the minimum error decision is based on the posterior probability of class membership *p*(*ω*_*i*_∣*x*) as [33]

(3) $$p(\omega_{i}|x)=p(\omega_{i})\frac{p(x|\omega_{i})}{p(x)}$$

where *p*(*x*∣*ω*_*i*_) is the class-conditional probability density function (PDF), *p*(*ω*_*i*_) is the prior probability, *p*(*x*) is the unconditional PDF, and *ω*_*i*_ denotes the *i*th class, *i*=1,2,…,*C*.

The discriminant function is defined as *g*_*i*_(*x*)=*log*[*P*(*ω*_*i*_)*p*(*x*∣*ω*_*i*_)],*i*=1,2,…,*C*. The class label of *x* is *ω*_*i*_, if *g*_*i*_(*x*)≥*g*_*j*_(*x*), for all *i*≠*j*. The common assumption is that all class-conditional PDF are the normal distribution with means *μ*_*i*_ and covariance matrices *Σ*_*i*_. The final decision rule can make use of the following discriminant function:

(4) $$g_{i}(x)=\;\text{log}\;(p(\omega_{i}))-\frac{1}{2}{\left(x-\mu_{i}\right)}^{T}\Sigma_{i}^{-1}(x-\mu_{i})$$

where the unbiased estimates of *μ*_*i*_ and *Σ*_*i*_ are defined as

(5) $$\begin{array}{l} \mu_{i}=\frac{1}{n_{i}}\underset{x\in \omega_{i}}{\sum }x\; \end{array}$$

(6) $$\begin{array}{l} \Sigma_{i}=\frac{1}{n_{i}-1}\underset{x\in \omega_{i}}{\sum }(x-\mu_{i}){\left(x-\mu_{i}\right)}^{T}\; \end{array}$$

It is shown that the discriminant function constructs the pairwise linear decision surface if all covariances *Σ*_*i*_ are the same as pooled within-class scatter matrix *Σ*_*W*_:

(7) $$\Sigma_{W}=\overset{c}{\underset{i=1}{\sum }}\frac{n_{i}-1}{n-c}\Sigma_{i};$$

where *n* is the total number of the EMG patterns. It is called the LDA classier. If *Σ*_*i*_ is assumed to be different, the decision boundaries are the hyperquadric surface and this is the QDA classifier. For sufficient data condition, QDA is superior to LDA since the specific covariance estimates accurately characterize the second-order information in the classification model and has nonlinear separability for different classes. Otherwise, LDA using the averaged pooled covariance controls less parameters and has better performance for small data condition.

### Self-enhancing method for classifiers

We extend the LDA and QDA classifiers to the self-enhancing versions (SELDA and SEQDA) using additional knowledge from the classified data in testing set. The parameters of the original classifiers are adjusted by updating the mean vector and covariance matrix. Suppose that there are *N* patterns used for training the classifier, and the new-coming testing EMG feature patterns are acquired as *x*_*N*+1_,*x*_*N*+2_,*x*_*N*+3_,…. To illustrate the proposed self-enhancing procedure, we make the case of the first testing *x*_*N*+1_ pattern updating as an example. Let the pattern *x*_*N*+1_ be *z* and labeled as the *k*th class by the original classifier, there are original *nc*_*j*_ patterns for each class before updating, where *j*=1,2,…,*C*. After the *z* pattern updating, the number of patterns in *k*th class becomes $nc_{k}^{\prime }=nc_{k}+1$.

The updated mean vector ${\tilde{\mu }}_{k}$ for the *k*th class is

(8) $${\tilde{\mu }}_{k}=\frac{nc_{k}*\mu_{k}+z}{nc_{k}+1}$$

Denote $S_{k}=\sum_{i=1}^{nc_{k}}(x_{i}-\mu_{k}){\left(x_{i}-\mu_{k}\right)}^{T}$ and ${\tilde{S}}_{k}=\sum_{i=1}^{nc_{k}+1}(x_{i}-{\tilde{\mu }}_{k}){\left(x_{i}-{\tilde{\mu }}_{k}\right)}^{T}$. $\sum_{i=1}^{nc_{k}}(x_{i}-\mu_{k})=0$. The relation between *S*_*k*_ and ${\tilde{S}}_{k}$ for the *k*th class is

(9) $$\begin{array}{l} {\tilde{S}}_{k}=\;\overset{nc_{k}+1}{\underset{i=1}{\sum }}(x_{i}-{\tilde{\mu }}_{k}){\left(x_{i}-{\tilde{\mu }}_{k}\right)}^{T}\\ \;=\;\overset{nc_{k}}{\underset{i=1}{\sum }}(x_{i}-{\tilde{\mu }}_{k}){\left(x_{i}-{\tilde{\mu }}_{k}\right)}^{T}+(z-{\tilde{\mu }}_{k}){\left(z-{\tilde{\mu }}_{k}\right)}^{T}\\ \;=\;\overset{nc_{k}}{\underset{i=1}{\sum }}(x_{i}-\frac{nc_{k}*\mu_{k}+z}{nc_{k}+1}){\left(x_{i}-\frac{nc_{k}*\mu_{k}+z}{nc_{k}+1}\right)}^{T}\\ \;+(z-\frac{nc_{k}*\mu_{k}+z}{nc_{k}+1}){\left(z-\frac{nc_{k}*\mu_{k}+z}{nc_{k}+1}\right)}^{T}\\ \;=\;S_{k}+\frac{nc_{k}}{{\left(nc_{k}+1\right)}^{2}}(z-\mu_{k}){\left(z-\mu_{k}\right)}^{T}\\ \;+\frac{nc_{k}^{2}}{{\left(nc_{k}+1\right)}^{2}}(z-\mu_{k}){\left(z-\mu_{k}\right)}^{T}\\ \;=\;S_{k}+\frac{nc_{k}}{\left(nc_{k}+1\right)}(z-\mu_{k}){\left(z-\mu_{k}\right)}^{T} \end{array}$$

The parameters of other classes are unchanged for the *z* pattern updating. Then, let $C_{k}=\frac{n_{k}}{\left(nc_{k}+1\right)}(z-\mu_{k}){\left(z-\mu_{k}\right)}^{T}$,

(10) $${\tilde{S}}_{k}=S_{k}+C_{k}$$

For the SEQDA classifier, the class covariance matrix ${\tilde{\Sigma }}_{k}$ is updated by

(11) $$\begin{array}{l} {\tilde{\Sigma }}_{k}=\frac{1}{nc_{k}+1}{\tilde{S}}_{k}\\ \;=\frac{1}{nc_{k}+1}S_{k}+\frac{1}{nc_{k}+1}C_{k}\\ \;=\frac{nc_{k}}{nc_{k}+1}\Sigma_{k}+\frac{1}{nc_{k}+1}C_{k} \end{array}$$

For the SELDA classifier, the pooled covariance matrix ${\tilde{\Sigma }}_{W}$ is updated by

(12) $$\begin{array}{l} {\tilde{\Sigma }}_{W}=\overset{c}{\underset{j=1}{\sum }}\frac{nc_{j}^{\prime }}{N+1}{\tilde{\Sigma }}_{j}\\ \;=\overset{c}{\underset{j=1,j\neq k}{\sum }}\frac{nc_{j}}{N+1}\Sigma_{j}+\frac{nc_{k}+1}{N+1}{\tilde{\Sigma }}_{k}\\ \;=\overset{c}{\underset{j=1}{\sum }}\frac{nc_{j}}{N+1}\Sigma_{j}+\frac{1}{N+1}C_{k}\\ \;=\frac{N}{N+1}\Sigma_{W}+\frac{1}{N+1}C_{k} \end{array}$$

The entire procedure of self-enhancing classifier works in two steps. First, the parameters of original classifier are initiated by the training set. Second, the trained classifier is evaluated by the testing set. The continuous classifier receives the EMG feature data and predicts the class labels for them. The proposed incremental self-enhancing method updates the parameters of the discriminant classifier immediately by above equations (9), (11) and (12) when the current EMG feature is classified to one output of the possible motions. Therefore, the information of testing data is continuously incorporated into the classification model. This sequential parameter updating is suitable for the continuous EMG PR in the real-world application. In addition, the self-enhancing automatically proceeds through the testing stage without manual operations.

### EMG data acquisition

**Figure 2:**
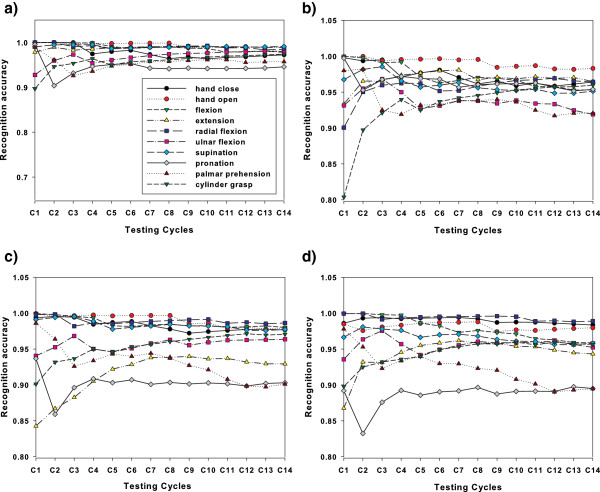
**Photo of electrode placements on the forearm.** (**a**) Posterior view: extensor carpi radials, extensor carpi ulnaris. (**b**) Anterior view: palmaris longus, flexor carpi ulnaris.

The experiment included ten classes of hand and wrist motions, which are pronation, supination, hand closing, hand opening, radial flexion, ulnar flexion, flexion, extension, palmar and cylinder grasp. We collected the EMG data using a portable EMG system (ME6000, Mega Electronics Ltd, Kuopio, Finland) with a band-pass filter of bandwidth 8–500 Hz and a 14 bit A/D converter. CMRR is Typ. 110 dB. The 1000 Hz sampling frequency was satisfactory for obtaining sufficient information on the surface EMG signal, as the most relevant information is contained in the range of 20–500 Hz. Two surface Ag/AgCl disc electrodes of one bipolar-electrode pair were placed 2 cm apart, after first rubbing the skin with alcohol. Four channels of surface EMG signals were used for the data acquisition, placed on palmaris longus, flexor carpi ulnaris, flexor digitorum supercifialis, extensor digitorum (shown in Figure 2). All recruited subjects have signed the informed consent. The procedures conformed to the Declaration of Helsinki. Ethical approval was obtained from the Bioethics Committee, School of Biomedical Engineering, Shanghai Jiao Tong University.

**Figure 3:**
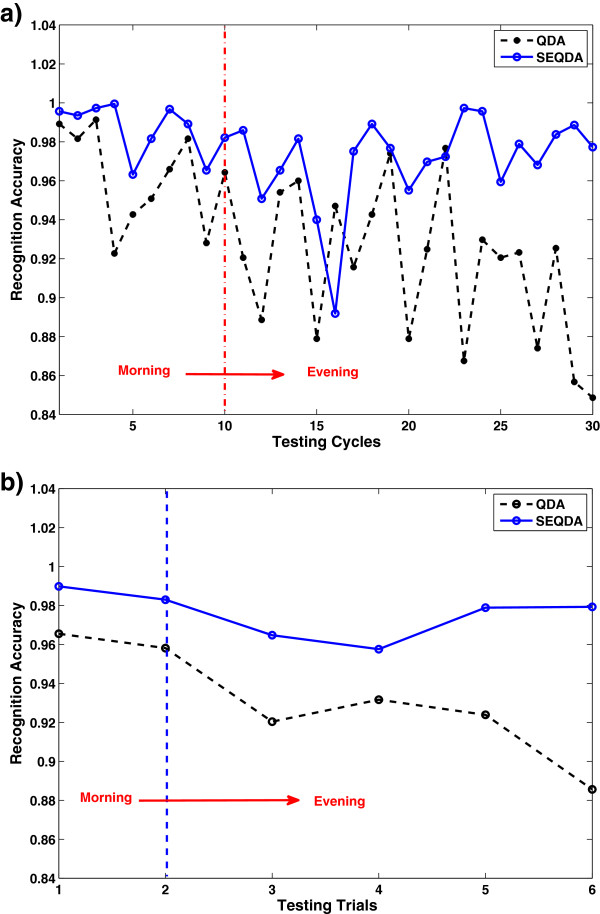
**Two protocols for EMG data collection.** Protocol 1: EMG data are collected during a short period (2 ∼3 hours) at one time. Protocol 2: EMG data are collected at two separate times during a day, and the time interval is about 6 ∼7 hours.

The EMG measurement was designed in two protocols as shown in Figure 3. In first protocol, the testing data and training data are collected at one time, i.e. there is no break between testing data measurement and training data measurement. This is the general case like most previous research. While, in the second protocol, the time scan is about 9 ∼11 hours. It is close to the real-world application situation, and it is the first try in this area.

In the first protocol, ten able-bodied subjects (seven males and three females) participated, and the age ranges from 22 to 28. Before the data collection, the instruction photographs of hand and wrist motions were shown to the participants. They could practice the desirable movements for a moment in order to be familiar with the experiment procedure. During the experiment, all participants naturally extended their arms toward to the ground, and performed each motion with natural force as that in their daily life (no need to use large force on purpose). In each cycle, the participants were instructed to sequentially perform ten motion classes. Each contraction was held for 5 s and separated by 5 s resting interval. The participant could relax every two cycles and no fatigue was reported. The experiment collected twenties cycles of ten motions for each participant. The whole experiment of data acquisition lasted for about 2 to 3 hours for each participant. In EMG PR evaluation, the first 6 cycles were assigned as a training set and the next 14 cycles as a testing set.

In the second protocol, four able-bodied healthy subjects (all males) participated and the age ranges from 22 to 25. The EMG data were acquired at two separate times for each subject in one day. One time was in the morning, and the other was in the evening. The time interval was about 6 ∼7 hours. During the interval, the EMG electrodes were not removed, and the subjects still could do the daily activities as usual. The other procedure of EMG measurement is the same as that in the first protocol. For each subject, 35 cycles of measurement were conducted (15 cycles in the morning, and 20 cycles in the evening), and each trials contained five cycles. The data of the first 5 cycles are used for training, while the data of rest 30 cycles are used for testing.

## Experiment results

Novel classifiers with self-enhancing are proposed, while the available feature extraction methods are not improved in this work. To evaluate the performance of self-enhancing classifiers, AR and FC feature sets are prepared, where the 6th order AR coefficients with RMS value of each channel form the AR feature, and the first seven FC coefficients of each channel construct the FC feature. The two feature sets are 24 dimensional vectors. For EMG feature extraction, the data from a 200 ms analysis window are used to estimate the feature, with the analysis window incremented by 25 ms. The traditional classifiers (LDA and QDA) and the proposed classifier (SELDA and SEQDA) are applied respectively, and their performance is compared. Please note that the results in subsections ‘Comparison of self-enhancing methods with the traditional classifiers, Effect of mean vector and covariance updating on the classification performance and Changes of recognition accuracy and classifier parameters across different testing trials’ are accomplished using the EMG data from the first protocol, and subsection ‘Evaluation on long-term EMG data’ will show the results using the data from the second protocol.

### Comparison of self-enhancing methods with the traditional classifiers

We compare the SELDA and SEQDA classifiers with their original versions using both AR and FC feature sets. The parameters of SELDA and SEQDA, such as class mean vectors, class covariances and pooled covariances, are updated using the testing data respectively. The LDA and QDA classifiers keep the original model learned by the training set. Table 1 lists the participant-specific and mean/standard deviation (std) recognition accuracy (RA) rates of different classifiers, where the combinations of the feature and classifier achieving the best performance for each participant are highlighted in bold. From this table, it can be observed that 1) the self-enhancing method can improve the classification performance when compared to the original classifiers. 2) the self-enhancing classifiers has less variability (small std) than their original versions and thus shows more robust performance. 3) the SEQDA method (about 2.2%) has greater performance improvement than the SELDA (about 1.6%) for both feature sets, indicating that individual class covariances updating is superior to the pooled covariance updating.

**Table 1:** Participant-specific and mean RA results of different classifiers

		**Classification accuracy(%)**			
**Participant**		**LDA**	**QDA**	**SELDA**	**SEQDA**
P1	AR	95.29	94.36	97.36	96.39
	FC	95.30	96.65	97.12	**99.29**
P2	AR	92.95	88.15	93.58	92.95
	FC	94.60	94.61	97.09	**97.60**
P3	AR	96.23	98.02	97.14	99.44
	FC	98.00	97.79	98.89	**99.62**
P4	AR	91.97	91.58	93.00	92.60
	FC	91.83	93.49	92.49	**94.26**
P5	AR	90.23	90.47	91.68	92.60
	FC	92.05	93.85	94.49	**98.10**
P6	AR	94.17	95.34	96.62	97.09
	FC	94.48	95.60	96.48	**98.15**
P7	AR	89.24	84.88	93.66	88.96
	FC	86.61	90.88	90.79	**94.53**
P8	AR	93.18	96.88	93.58	98.33
	FC	95.59	97.45	98.22	**98.54**
P9	AR	96.75	96.32	98.24	97.18
	FC	97.25	98.17	98.22	**99.05**
P10	AR	95.66	95.31	96.20	**97.64**
	FC	95.08	96.17	95.45	97.04
	AR	93.57 ±	93.13 ±	95.11 ±	95.34 ±
Mean ± std		2.54	4.24	2.25	3.32
	FC	94.08 ±	95.47 ±	95.67 ±	97.62 ±
		3.26	2.28	2.52	1.87

The best RA results for each participant are highlighted in bold.

We have also studied the mean RA results of individual motions. For the prosthesis control, the reliability of systems requires high accuracy not only within the mean RA rate but also within the RA of each motion. The poor recognition of certain specific motions would be of hazard to the safe operation of prostheses. It is found that the self-enhancing method raises RA results for most motions. For SEQDA + FC method, the RAs of motions are all above 93%.

### Effect of mean vector and covariance updating on the classification performance

The self-enhancing mechanism is realized by two types of updating, the class mean vectors and the class (or pooled) covariances, which respectively characterize the first order and second order information in the LDA and QDA classifiers. This experiment aims to evaluate how these parameters impact on the RA results. The SELDA (M) or SEQDA (M) and SELDA (C) or SEQDA (C) denote the mean vectors updating and covariances updating respectively. Table 2 lists the participant-specific and mean classification accuracies of different classifiers. It shows that each parameter updating has the positive effect for improving the classification performance. The mean results of SEQDA(C) and SEQDA (M) are improved by 2.2% and 0.75% for AR, and 1.99% and 1.1% for FC respectively when compared to QDA. The mean results of SELDA(C) and SELDA (M) are improved by 0.48% and 1.55% for AR, and 0.67% and 1.22% for FC respectively when compared to LDA.

**Table 2:** Participant-specific and mean RA results of the different parameter updating for SEQDA and SELDA

		**Classification accuracy(%)**			
**Participant**		**SELDA (M)**	**SELDA (C)**	**SEQDA** **(M)**	**SEQDA** **(C)**
P1	AR	96.98	96.39	95.52	96.26
	FC	96.48	96.10	97.56	99.18
P2	AR	93.56	93.14	88.78	92.84
	FC	96.28	95.19	95.69	97.45
P3	AR	97.32	96.20	98.59	99.44
	FC	98.87	98.04	98.60	99.51
P4	AR	92.95	92.15	92.03	92.63
	FC	92.18	92.31	93.87	94.27
P5	AR	92.37	89.78	91.25	93.58
	FC	93.97	92.80	95.07	97.90
P6	AR	96.61	94.85	95.83	97.00
	FC	96.10	95.46	97.06	97.91
P7	AR	93.72	91.64	85.45	88.76
	FC	90.16	89.37	93.09	94.59
P8	AR	93.97	93.14	97.55	98.03
	FC	95.87	95.49	98.11	97.70
P9	AR	97.58	97.48	96.78	97.14
	FC	97.78	97.80	98.24	99.00
P10	AR	96.18	95.72	95.98	97.59
	FC	95.33	94.93	96.53	97.13
Mean	AR	95.12	94.05	93.87	95.33
	FC	95.30	94.75	96.38	97.46

### Changes of recognition accuracy and classifier parameters across different testing trials

**Figure 4:**
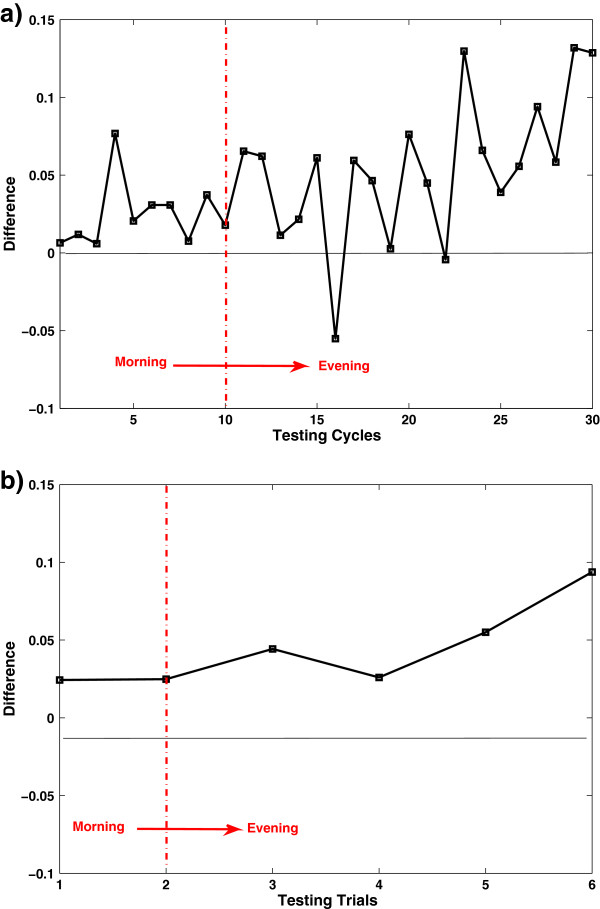
**Mean recognition accuracy across testing cycles.** (**a**) Recognition accuracy of different classifiers in the testing cycles using AR features, (**b**) Recognition accuracy of different classifiers in the testing cycles using FC features.

**Figure 5:**
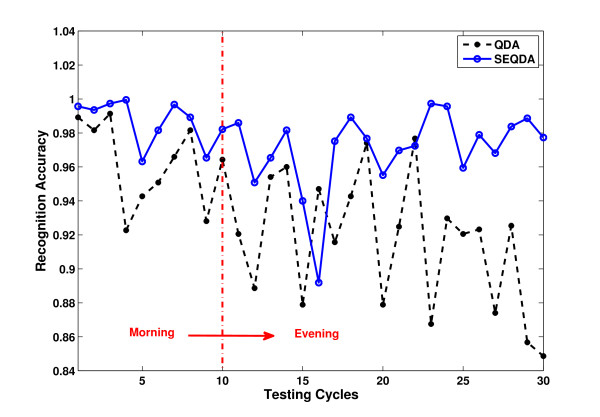
**Recognition performances of ten motion classes across testing cycles.** The motion classes include pronation, supination, hand closing, hand opening, radial flexion, ulnar flexion, flexion, extension, palmar and cylinder grasp. (**a**) and (**b**) show the performance of SEQDA using FC and AR features respectively. (**c**) and (**d**) show the performance of SELDA using FC and AR features respectively.

To compare the recognition performance of the self-enhancing and original classifiers across the testing stage, we plot Figure 4 displaying the mean RA results for each testing cycle, where the *i*th mean RA averages the classification results over the past *i* testing cycles, and the final result is the overall mean RA. These plots show that the RA rates of the classifiers change over time (testing cycles), and the final RA rates of the original classifiers are lower than their preceding rates. Figure 5 presents the RA performance based on SEQDA and SELDA for ten motion classes across testing cycles.

We have investigated the changes of some classifier parameters across different testing cycles. Under the assumption of data with a Gaussian distribution, the class mean vectors *μ* and the covariances *Σ* of the discriminant classifier describe the distribution of each class by a hyperellipsoid. The class mean vectors indicate the difference between classes, and the covariances depict the shape of distributions referring to equations (5),(6). The principal axes of these hyperellipsoids are given by the eigenvectors of the covariances, and the eigenvalues determine the lengths of these axes [34]. To describe the direction changes of principal axes and mean vectors, the cosine of angle between the original vector (the training one) and the current vector (the *i*th testing cycle) is given by

(13) $$\text{cos}=\frac{v_{0}\cdot v_{i}}{\left|v_{0}||v_{i}\right|}$$

where *v*_0_ and *v*_*i*_ denote the original and current vectors respectively, · denotes the internal product, and |*v*| denotes the norm of the vector.

Based on the FC feature, we study the changes on SEQDA and SELDA for a specific subject (P6) respectively. The four kinds of parameters are further considered: length of class mean vectors, length of first two principal axes of class covariances, cosine of angle of class mean vectors, and cosine of angle of first two principal axes of class covariances. All the parameters more or less show some changes in different testing cycles along the time, but there is no very significant and useful information. We can only find that the changes on pooled covariance of SELDA, the class mean vectors of both SELDA and SEQDA are relatively small. So perhaps they make minor contribution to adaptivity of the proposed classifier.

### Evaluation on long-term EMG data

In this part, based on the EMG data collected in the second protocol mentioned in EMG data acquisition section, we tested the performance of the proposed classifier. As QDA (SEQDA) generally performs better than LDA (SELDA), we just present the results on QDA (SEQDA). Only FC is used as the EMG feature here.

**Figure 6:**
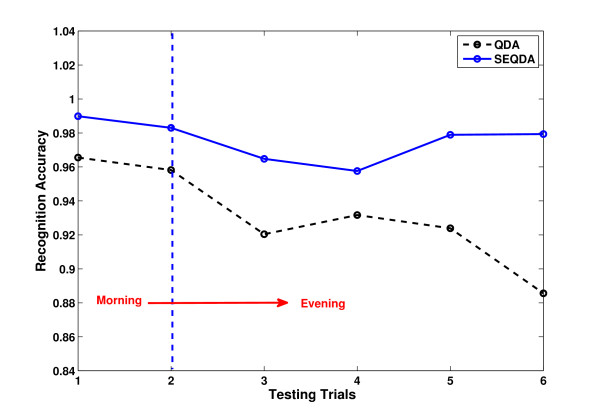
**Recognition accuracy for the individual subject (S1).** (**a**) Results of QDA/SEQDA in 30 testing cycles. (**b**) Results of QDA/SEQDA in 6 testing trials. Note: Each trial contains 5 cycles.

The results on RA of QDA and SEQDA for the four subjects are shown in Table 3. It is obvious that the general performance of SEQDA (97.58%) is 3.15% better than that of QDA (94.43%). Without loss of generality, we select the result of a subject (S1) to observe the change of classification accuracy along at different time points. In comparison, the results on average RA of 30 testing cycles and 6 trials (each trial contains 5 cycles) using QDA and SEQDA are illustrated in Figure 6. We can see the details from results represented in cycles, and find the general trend from results represented in trials.

**Table 3:** Average recognition accuracy of 10 types of motions on four subjects (S1-S4) using long-term EMG data

	**S1**	**S2**	**S3**	**S4**	**Mean**
QDA	93.45	93.09	94.33	96.85	94.43
SEQDA	95.56	97.56	97.63	99.58	97.58

**Figure 7:**
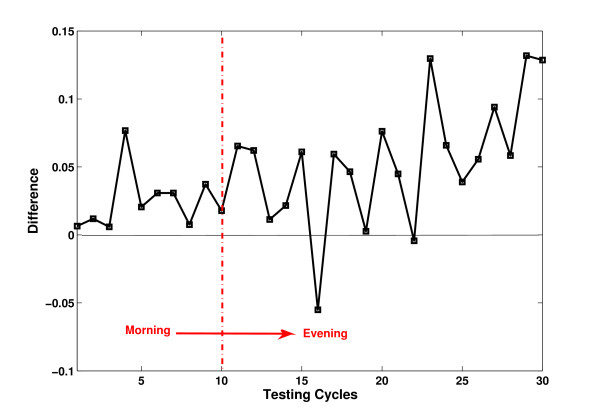
**Difference between QDA and SEQDA for the individual subject (S1).** (**a**) Results in 30 testing cycles. (**b**) Results in 6 testing trials. Difference represents the average RA of SEQDA minus that of QDA. Note: Each trial contains 5 cycles.

For a clear view, the difference of average RA between QDA and SEQDA (RA of SEQDA minus RA of QDA) is shown in Figure 7. The trend that SEQDA outperforms QDA can be observed. At the early stage, the difference is very small, while the difference becomes significant after certain time.

## Discussion

For feature sets in Table 1, FC shows better performance than AR when using the QDA. The possible reason is that covariances of FC vary from different classes and has nonlinear feature distribution. Therefore, nonlinear classifier such as QDA can better discriminate it. In the experiment, the FC feature presents better performance than the AR feature. A paired t-test [35] is employed to examine the statistical significance of the improvement by the use of self-enhancing method. The SEQDA significantly outperforms the QDA in the statistical test using both AR and FC features (p < 0.01). The SELDA is also significantly better than the LDA using both AR and FC features (p < 0.01). In addition, from Table I, we find that the FC+SEQDA is determined as the best combination of the featre and classifier for nine out of ten participants. AR+LDA is widely considered as a benchmark EMG classification method due to its good performance [8,24]. The proposed FC+SEQDA has the RA rate roughly 4% higher than AR + LDA.

In Table 2, for SEQDA, the class covariance updating presents greater improvement than the class mean vectors updating. On the contrary, the pooled covariance updating has less improvement than the class mean vectors updating for SELDA. The class mean vectors updating has different classification strength on SELDA and SEQDA. This might be caused by the different effects of the two (class or pooled) covariance estimates upon the classification performance. The combination of mean vector and covariance updating can further increase the RA results except the SELDA classifier using the AR feature. The paired t-test shows that the RA results are significantly improved when using the SEQDA(C), SEQDA(M) and SELDA(M) for both AR and FC features. The improvement of SELDA(C) is not significant. In a word, the class covariance updating and the class mean updating play major roles in both SEQDA and SELDA classifiers.

In Figure 4, RA rates of the traditional classifiers (LDA and QDA) decreases obviously. The reason of this RA decrease can be attributed the unobserved changes of experiment condition in 2–3 hours, including perspiration, humidity, cognitive intent variations or contraction intensity changes, soft tissue fluid fluctuations (slight spatial change) and so on. Perhaps, the experimental participants already have slight fatigue but they cannot exactly feel it, so they did not report it. It can be found that the RA differences of the two types of classifiers are enlarged with the increase of testing cycles. This may be attributed to the fact that the self-enhancing method can incorporate more information from testing set to the initial models and can accurately estimate the parameters of classifiers with change of the different testing cycles. It is observed that the performance declines in Figure 4 for all but the FC+SEQDA. It means that FC+SEQDA might be more robust than other combinations. In the experiment, the length of testing cycles might not be long enough for the declining of FC+SEQDA.

Figure 5 shows the classed-based performance for ten motion classes, where the classification performance of most motion classes declined with the increase of testing cycles. But a few classes (e.g. extension and cylinder grasp) have the increasing performance. The possible reasons for this phenomenon may include: 1) the adaptation enlarges the data size for training and therefore leads to more accuracy estimation of classifier parameters, particularly with covariance. 2) the training data have much difference from the first two or three testing cycles. The adaptation mechanism allows the classifier to learn the information in testing data and enhance the performance.

Regarding the evaluation on long-term EMG data as the results shown in Figure 6. The RA of QDA degrades obviously, which indicates the traditional QDA without adaptation cannot guarantee stable performance in a long duration. For SEQDA, the performance does not degrade much in general, although the performance is not good at several points. This is reasonable, because none can get absolutely perfect information from the testing data, and there must be some unexpected disturbing data. However, even the worst case of SEQDA (RA=0.885) is still better than any result of QDA.

The experimental evaluation confirms the efficiency of the proposed self-enhancing approaches. The SELDA and SEQDA classifiers outperform the original versions using both AR and FC features. The adaptation of classifier parameters has meaning at two levels. First, it can incorporate the information of testing data into the classifier. Second, it indeed enlarges the data for training the classifier. We think that these two adaptation factors will mutually improve the classification performance. The results also show that SEQDA is superior to SELDA and suggest the individual class covariance updating can give more accurate estimation of the second order information than the pooled covariance. The possible reason is that the class covariance updating takes the individual class information into accounts and thus is a type of semi-supervised method (using the classified labels), and the pooled covariances updating is an unsupervised method. The self-enhancing method provides the feedback on each testing EMG data to update the classification algorithm. Using online testing feedback of the current state of the prostheses will help the users to recognize the misclassification and to adjust themselves to proper conditions. It is expected that the two types of feedbacks, one to algorithms and another to users, will mutually improve the classification performance further. Moreover, similar to their original classifiers, SELDA and SEQDA have no hyperparameters and require no time-consuming trial-and-error procedures, facilitating their application to the prosthesis control.

Computational efficiency is an important implemental issue of the classification method. In our EMG PR algorithm, the AR model is estimated by the Burge algorithm, and the FC coefficients are computed by the fast algorithms such as FFT and DCT. The experimental hardware platform is a personal computer, consisted of a Core2 Duo 2.0G Hz CPU, 2G DDR2 memory. The software platform is Matlab version 7.1 under the windows XP operating system. To process 200 samples EMG data, the time cost of AR feature set is about 4 ms, the time cost of FC feature set is about 2 ms and the classifier requires 1 ∼2 ms. The FC feature extraction has relatively faster computing speed than AR by the use of fast algorithms. In addition to the original classification procedure, our self-enhancing method needs additional step to update the parameters of the classifier and it cost about 2 ms. More sophisticated digital signal processing hardware will expedite the online processing. Moreover, the self-enhancing method stores the class mean vectors, class covariances and pooled covariance for saving the model information after each updating and has no need to store the large EMG data.

The most promising highlight of the self-enhancing method is for the long-term EMG PR task, since it provides a basis for prosthetic control in real-world application. Our method continuously adds the immediate information of the EMG pattern to the classifier by updating the model parameters. The measurements involve the EMG data for about 8 ∼10 hours that may include the possible variation factors. The testing data have larger size than the training data and the ratio is 7:3. The results have verified the performance of adaptive ability of the proposed algorithm. It can be seen that RA results of SEQDA outperforms QDA in the testing stage especially in the late stage. The good RA results of self-enhancing classifiers exhibit their robust characteristics for long-term application. Actually, there are many factors contributing to the nonstationary changes of long-term EMG signals such as electrode position, muscle fatigue, or other physiological/psychological condition [25-27]. The underlying physiological mechanism needs more investigation, and this work does not focus on this issue. Evaluating on the longer-term EMG data such as over days and months may shed more lights on the self-enhancing approach into practice.

## Conclusion

In summary, this paper proposes a self-enhancing method for EMG classification based on the traditional LDA and QDA classifiers, which can incorporate the useful information of EMG signal in testing data to the classification model. The improved classifiers named as SELDA and SEQDA continuously update their parameters such as the class mean vectors, the class covariances and pooled covariances using the labelled EMG feature data. We have shown that the self-enhancing classifiers significantly improve the recognition performance of the EMG PR system including the preliminary application on long-term EMG data.

## Competing interests

The authors declare that they have no competing interests.

## Authors’ contributions

XC participated in algorithms, data recordings, data processing and drafted the manuscript. DZ conceived of the study, participated in algorithms, protocol of experiments, and drafted the manuscript. XC and DZ are the first co-authors. XZ coordinated the study, participated in its design and helped to polish the manuscript. All authors read and approved the final manuscript.
